# Supplementary material for: Evolution of transient RNA structure-RNA polymerase interactions in respiratory RNA virus genomes
Source: bioRxiv. 2023 Aug 2:2023.05.25.542331. Originally published 2023 May 26. Preprint. [Version 2] doi: 10.1101/2023.05.25.542331 (PMC10245964; doi:10.1101/2023.05.25.542331)
Supplement: Supplement 3 [file media-3.pdf]

Supplemental figures for:

Evolution of transient RNA structure-RNA polymerase interactions in respiratory RNA virus genomes

Charlotte Rigby[1,2,3], Kimberly Sabsay[1,4], Karishma Bisht[1], Dirk Eggink[5,6], Hamid Jalal[3], Aartjan J.W. te Velthuis[1,*]

[1] Lewis Thomas Laboratory, Department of Molecular Biology, Princeton University, 08544 New Jersey, United States.
[2] University of Cambridge, Department of Pathology, Addenbrooke's Hospital, Cambridge CB2 2QQ, United Kingdom
[3] Public Health England, Addenbrooke's Hospital, Cambridge CB2 2QQ, United Kingdom
[4] Sigler Institute, Princeton University, Princeton, NJ 08544, United States.
[5] Department of Medical Microbiology, Amsterdam UMC, Amsterdam, The Netherlands
[6] Center for Infectious Disease Control, National Institute for Public Health and the Environment (RIVM), Bilthoven, the Netherlands

* Address correspondence to: aj.te.velthuis@princeton.edu

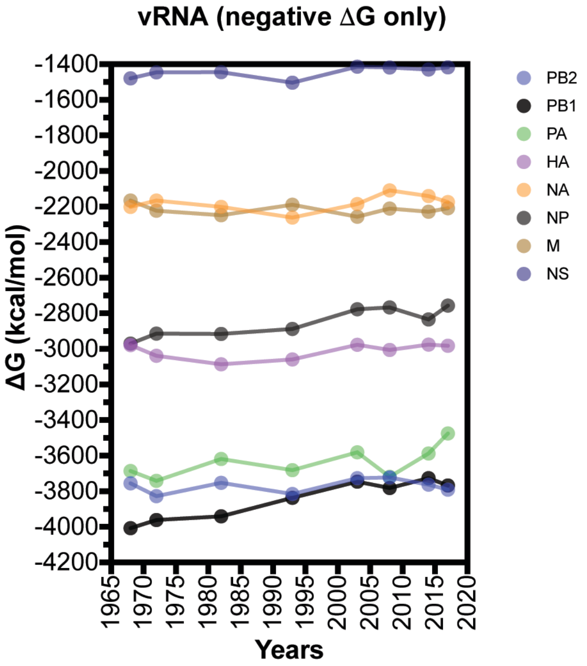

Figure S1. Analysis of t-loop free energy for IAV H3N2 isolates between 1968 and 2017 using negative free energy values only.

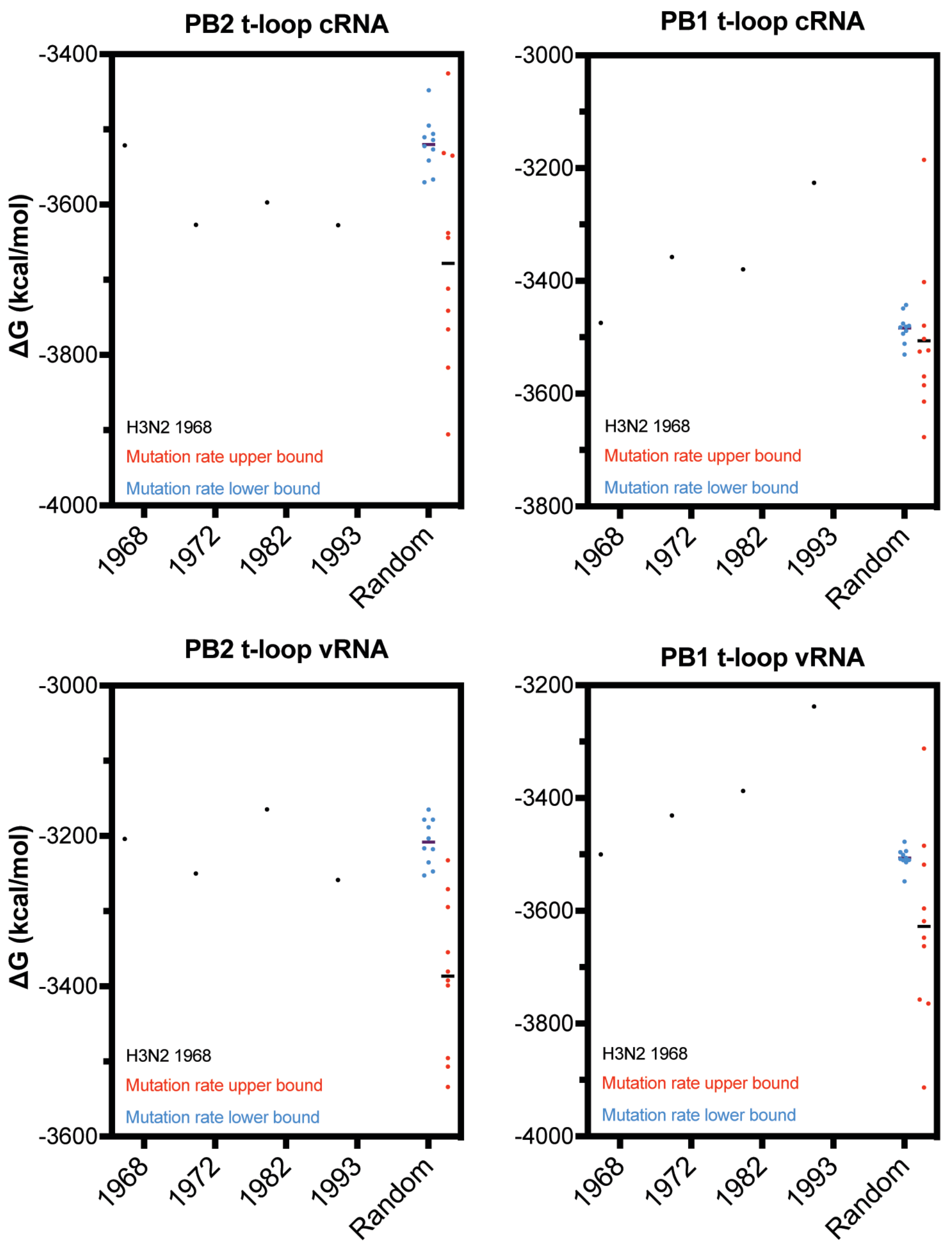

Figure S2. In silico mutation of IAV genome segments. Comparison of the t-loop free energy in the IAV PB2 and PB1 encoding genome segments (vRNA sense) following random mutation (in cRNA sense to preserve codon information).

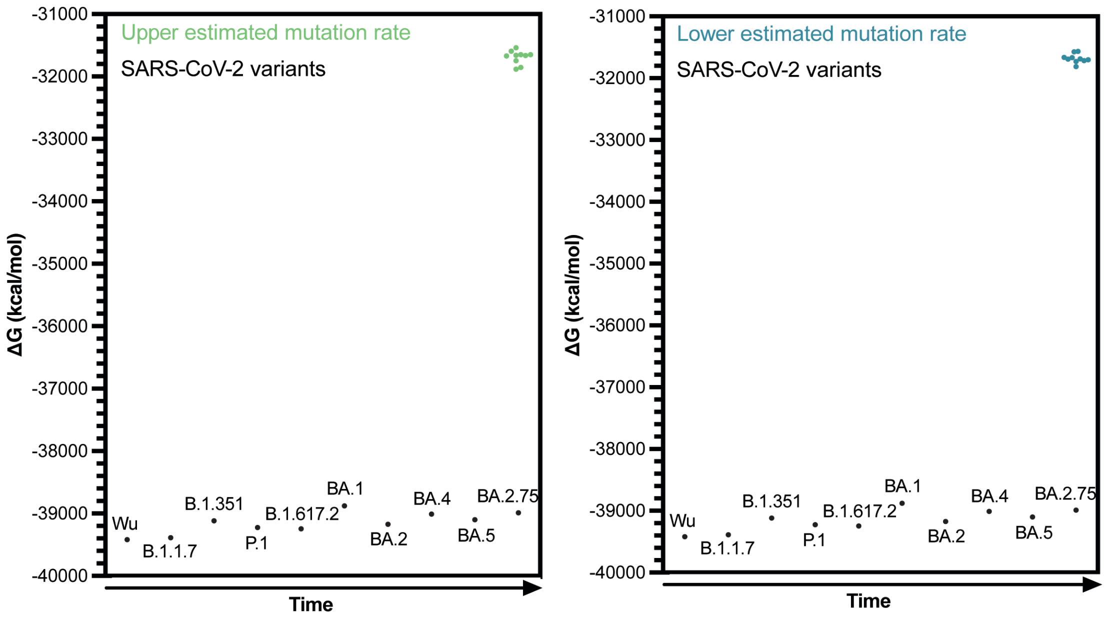

Figure S3. In silico mutation of SARS-CoV-2 genome. Comparison of the t-loop free energy in the SARS-CoV-2 genome following random mutation at two different rates of the Wuhan strain relative to the mean observed t-loop free energy in SARS-CoV-2 isolates.
